# Is peripheral paraneoplastic neurological syndrome possible in primary brain tumors?

**DOI:** 10.1002/brb3.465

**Published:** 2016-04-27

**Authors:** Magdalena Koszewicz, Slawomir Michalak, Malgorzata Bilinska, Slawomir Budrewicz, Mikolaj Zaborowski, Krzysztof Slotwinski, Ryszard Podemski, Maria Ejma

**Affiliations:** ^1^ Department of NeurologyWroclaw Medical UniversityBorowska 21351‐669WroclawPoland; ^2^ Department of Neurochemistry and NeuropathologyPoznan University of Medical SciencesPoznanPoland; ^3^ Neuroimmunological UnitMiroslaw Mossakowski Medical Research Center of the Polish Academy of SciencesWroclawPoland; ^4^ Division of Gynecologic OncologyDepartment of Gynecology, Obstetrics and Gynecologic OncologyPoznan University of Medical SciencesPoznanPoland

**Keywords:** Antineural antibodies, neurography, onconeural antibodies, paraneoplastic syndrome, peripheral neuropathy, primary brain tumor

## Abstract

**Introduction:**

Systemic malignant diseases cause the induction of autoimmunity, for example, paraneoplastic syndromes. There are no proofs of paraneoplastic syndromes in primary brain tumors. The aim of the study was to evaluate the involvement of the peripheral nervous system, together with an assessment of onconeuronal and antineural antibodies as indicators of humoral immune response against nervous system in patients with primary brain tumors.

**Materials and Methods:**

Clinical examinations, electrophysiological studies of peripheral nerves (motor and sensory conduction velocity studies, conduction velocity distribution tests, thermal and vibratory quantitative sensory tests, and sympathetic skin response tests) and muscles, blood sampling collection (assessment of onconeuronal, and antineural antibodies) were performed on 33 patients with newly recognized primary brain tumors within 2–4 days after their admission to our department.

**Results:**

We revealed statistically significant changes of peripheral nerves, more pronounced in the peroneal nerve in standard and conduction velocity distribution tests, as well as in sympathetic skin responses. We revealed significantly higher vibratory thresholds, and pain thresholds for cold and warm in the upper and lower limbs in the study group than in the controls. In five patients, we have identified anti‐neuroendothelium, anti‐GFAP, anti‐MAG, anti‐PCNA, and anti‐Ro52 antibodies.

**Conclusions:**

In patients with primary brain tumors, electrophysiological changes in peripheral nerves, together with the presence of the antineural antibodies suggest an autoimmune humoral response, and make the diagnosis of paraneoplastic neurological syndrome possible.

## Introduction

PNSs (Paraneoplastic neurological syndromes) are the remote effects of malignancy on the central and peripheral nervous system. They could have typical or nontypical clinical appearances (classical and nonclassical PNSs), and onconeuronal antibodies are thought to have a crucial role in their diagnosis (Michalak et al. 2009; Graus and Dalmau 2012). Malignant diseases outside the central nervous system cause the induction of autoimmunity, and therefore many other antibodies could be found in these cases, among them antibodies detected in autoimmune rheumatic diseases (antinuclear antibodies, rheumatoid factor, antiphospholipid antibodies) (Abu‐Shakara et al. 2001; Michalak et al. 2009; Smeenk 2009). PNSs are typically present in the course of different types of cancer localized outside the central nervous system. There are very few case reports (Barisic et al. 2007; Derrett‐Smith and Isenberg 2008; Sanli et al. 2010; Melguizo et al. 2011; Nakano et al. 2013) of possible paraneoplastic syndromes associated with primary brain tumors.

The aim of the study was to evaluate the involvement of the peripheral nervous system, together with an assessment of onconeuronal and antineural antibodies as the indicators of humoral immune response against nervous system in patients with primary brain tumors.

## Materials and Methods

The study was approved by the local Bioethics Committee at Wroclaw Medical University. All patients gave informed written consent to participate in the study.

We included 33 patients (20 men, 13 women), mean age 53.0 ± 15.2 years old, with newly diagnosed primary brain tumors. We excluded all patients with a history of rheumatic diseases, diabetes mellitus, polyneuropathy, myopathy, thyroid function impairment, vitamin deficiency, and all diseases which could influence the peripheral nervous system and muscles, together with workers with chronic toxin exposure, and those addicted to alcohol and drugs. The control group consisted of 43 healthy (without any disorders influenced the peripheral nervous system) volunteers (students, colleagues), sex, and age matched (24 men, 19 women, 51.7 ± 10.1 years old).

Electrophysiological studies and blood sampling collection were performed on all patients within 2–4 days after their admission to our department.

Standard motor and sensory conduction studies were performed in the ulnar and peroneal nerves contralaterally to the hemiparesis with distal latency, amplitude, and conduction velocity estimation. CVD (Conduction velocity distribution) tests were performed in the same nerves, lower and upper quartiles, median, and the dispersion of conduction velocity between lower and upper quartiles was calculated. Thermal (sensation and pain thresholds for cold and warm temperatures) and vibratory QST (quantitative sensory tests) were performed in C8 and L5 regions. SSR (Sympathetic skin responses) for electrical stimuli were assessed from hand and foot. On the same side, we evaluated biceps and tibialis anterior muscle function; amplitude, area, duration and polyphasia of motor unit action potentials, and amplitude and density of maximal effort patterns were analyzed.

Indirect immmunofluorescence with monkey cerebellum, peripheral nerve, pancreas, and intestine as substrates (EUROIMMUN, Luebeck, Germany) was performed on all patients to assess onconeuronal antibodies: anti‐Hu, anti‐Yo, anti‐Ri, antiy‐CV2, antiy‐Ma/Ta, anti‐amphiphysin, and antineuronal antibodies: anti‐GAD, anti‐MAG, anti‐neuroendothelium, anti‐peripheral myelin, anti‐GFAP. The presence of the antibodies and the borderline cases were confirmed by line blot with Hu, Yo, Ri, CV2, Ma/Ta, amphiphysin recombinants (EUROIMMUN, Germany). The EUROIMMUN company establishes and applies a quality management system that fulfills the requirements certified by EN ISO 9001:2008 (The certificate is valid until 2017). The tests obtained also with “Conformite Europeenne” certificate (CE Mark). EUROIMMUN warrants design, development, production, and services of immunofluorescence systems for in vitro diagnosis in humans (Certificate of TÜV Rheinland Products GmbH‐ registration number SY 60076866 0001). Moreover, Department of Neurochemistry and Neuropathology participates in external quality control for indirect immunofluorescence and line blot and was certified by Institut fur Qualitatssicherung, Germany (certificate QV 1111‐15013, II/2015).

When positive reaction with nucleosome antigens was observed, the patients' serum was retested with line blot, based on ANA Profile 5 (EUROIMMUN, Germany). According to the nomenclature introduced by Graus et al. (2004) onconeuronal antibodies (anti‐Hu, anti‐Yo, anti‐Ri, antiy‐CV2, antiy‐Ma/Ta, anti‐amphiphysin) identified by means of two – step method are “well‐defined”. Antineural are antibodies that react with nervous tissue (cerebellum, peripheral nerve) antigens in indirect immunofluorescence test. All the tests used for analyses enabled the detection of IgA + IgM + IgG antibodies.

In the statistical analysis, we worked out the number of cases (N), mean values (X), median (M), range (min–max), lower and upper quartiles (25Q, 75Q), and SD (standard deviation) of the continuous parameters. We verified the assumption of the equality of means in the subsequent samples via ANOVA variance analysis and for the composite variance group via the nonparametric Kruskal–Wallis test (homogeneousness of variance was checked with Bartlett's test). *P* ≤ 0.05 was considered significant. We used the statistical software program EPIINFO 7.1.1.14 (Centers for Disease Control and Prevention, Atlanta, GA).

## Results

Glioblastoma multiforme was diagnosed in 15 of our patients, astrocytoma fibrillare grade II in five, ependymoma in three, astrocytoma anaplasticum in three, schwanoma, meningioma, meningioma pseudomonas, medulloblastoma, neurilemmoma, astrocytoma pilocyticum, and oligodendroglioma anaplasticum – in one each. In 23 patients, the tumor was localized in the left hemisphere, in 10, in the right. The degree of disability due to hemiparesis was between two and four points in the Rankin scale. In the neurological examination, the clinical symptoms of polyneuropathy were found in three patients (sensory impairment in “gloves and socks” distribution, lack of Achilles reflexes in one patient). There were no other abnormalities in their neurological status. The comparison between the patients and controls revealed statistically significant changes of peripheral nerves, more pronounced in the peroneal nerve. The amplitude and the motor conduction velocity were significantly lower in the patient group, and the CVD test confirmed the lowering of the conduction velocities in the upper and lower quartiles and median. The dispersion of conduction velocity between lower and upper quartiles was significantly bigger in the patient group. The latency and amplitude in the sural nerve were also significantly lower in the patient group in comparison with the controls (Table 1), as well as SSR amplitudes from hand and foot (Table 2). The latencies of SSR were prolonged, but were only statistically significant in the foot. Additionally, we revealed significantly higher vibratory thresholds and pain thresholds for cold and warm from upper and lower limbs in the study group than in the controls (Table 3). Statistical analysis of standard muscle parameters did not reveal any significant differences.

**Table 1:** Standard motor conduction parameters (latency (msec), amplitude (mV), conduction velocity (m/sec)), and CVD (lower, upper quartile, median velocity, and CVD diversity) in peroneal nerve

Peroneal nerve	Patient group (*n* = 33)	Control group (*n* = 43)	*P*
Latency (msec)	4.28 ± 0.97	4.32 ± 0.61	0.385
Amplitude (mV)	5.79 ± 2.08	7.84 ± 1.53	<0.0001
Conduction velocity (m/sec)	45.3 ± 4.7	49.6 ± 4.1	<0.0001
CVD lower quartile (m/sec)	36.3 ± 4.7	40.7 ± 2.5	<0.0001
Median velocity (m/sec)	40.4 ± 4.5	45.0 ± 2.8	<0.0001
CVD upper quartile (m/sec)	42.9 ± 4.5	48.4 ± 2.6	<0.0001
CVD diversity (m/sec)	6.59 ± 2.69	7.65 ± 1.15	0.0017

CVD, conduction velocity distribution.

**Table 2:** Standard sensory conduction test (latency (ms), amplitude (mV), and conduction velocity (m/sec)) in sural nerve

Sural nerve	Patient group (*n* = 33)	Control group (*n* = 43)	*P*
Latency (msec)	3.01 ± 0.52	2.76 ± 0.35	0.023
Amplitude (mV)	13.9 ± 10.7	19.3 ± 7.4	0.012
Conduction velocity (m/sec)	45.5 ± 16.0	51.8 ± 4.3	0.093

**Table 3:** SSR (latency (msec), amplitude (*μ*V)) from hand and foot

SSR	Patient group (*n* = 33)	Control group (*n* = 43)	*P*
Hand latency (msec)	1541.4 ± 214.6	1527.4 ± 118.6	0.090
Hand amplitude (*μ*V)	1380.5 ± 1004.5	2810.3 ± 1739.4	0.0002
Foot latency (msec)	2192.3 ± 3001.0	2156.3 ± 278.6	0.0345
Amplitude (*μ*V)	548.0 ± 476.3	1829.8 ± 1201.1	<0.0001

SSR, Sympathetic skin responses.

**Figure 1:**
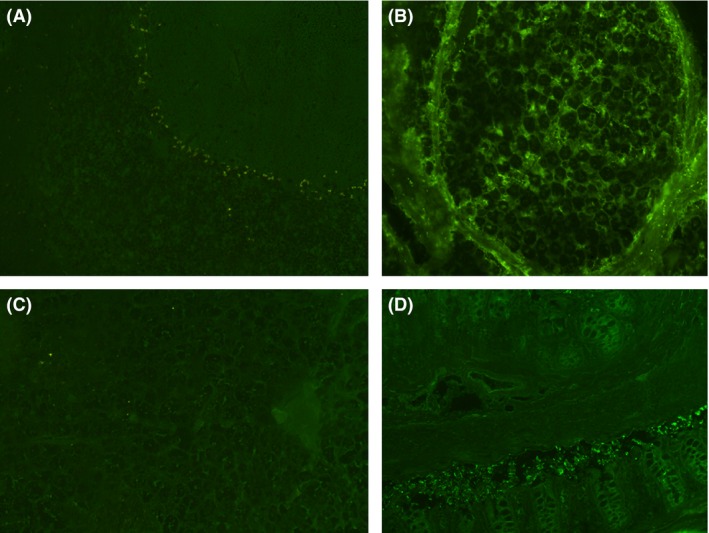
Negative results of indirect immunofluorescence. (A) monkey cerebellum, (B) monkey peripheral nerve, (C) monkey pancreas, (D) monkey intestine.

**Figure 2:**
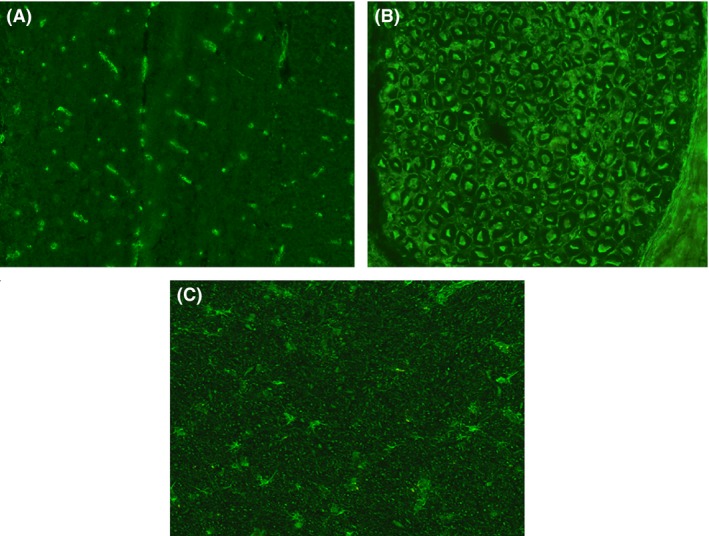
Anti‐ neuroendothelium antibodies (A), anti‐MAG antibodies (B), anti‐GFAP antibodies (C).

**Figure 3:**
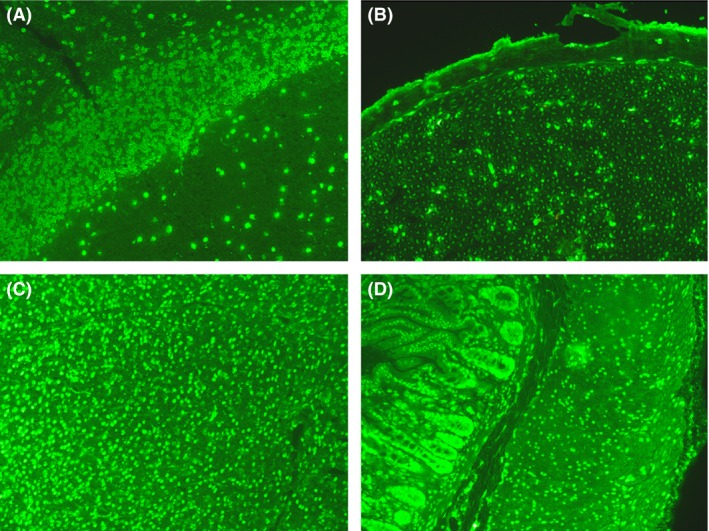
Antinucelosome antibodies. Positive reaction with monkey cerebellum (A), monkey peripheral nerve (B), monkey pancreas (C), monkey intestine (D).

In the screening tests, we did not find any onconeuronal antibodies at all. Negative results for the reaction with monkey cerebellum and peripheral nerve are presented in Figure 1(A and B, respectively). Antineural antibodies were present in 10 patients; and this included antinucleosome antibodies in seven, against neuroendothelium in one (Fig. 2A), anti‐MAG in 1 (Fig. 2B), and anti‐GFAP in 1 (Fig. 2C). Line blot confirmed the presence of antinucleosome antibodies in two cases detected by indirect immunofluorescence (Fig. 3), and these were: anti‐PCNA and anti‐Ro52. Thus, none of the well‐defined onconeural antibodies were found in our patients. The statistical analysis between groups of patients with and without antibodies did not show any significant differences. The electrophysiological findings in five patients with confirmed antibodies indicated the presence of neuropathy, but only one, with anti‐MAG antibodies, had clinically obvious polyneuropathy (Table 4).

**Table 4:** Sensation and pain thresholds for cold and warm temperatures (°C), and vibratory threshold (*μ*) in QST performed in C8 and L5 regions

QST Thresholds hand	Patient group (*n* = 33)	Control group (*n* = 43)	*P*	QST Thresholds foot	Patient group (*n* = 33)	Control group (*n* = 43)	*P*
Cold sensation (°C)	29.3 ± 1.9	29.6 ± 0.6	0.863	Cold sensation (°C)	26.7 ± 2.5	27.1 ± 1.6	0.945
Warm sensation (°C)	34.5 ± 0.9	34.4 ± 0.7	0.519	Warm sensation (°C)	38.9 ± 3.5	37.9 ± 2.16	0.470
Pain for cold (°C)	18.7 ± 6.1	24.9 ± 1.8	<0.0001	Pain for cold (°C)	18.3 ± 6.6	24.0 ± 2.0	0.0005
Pain for warm (°C)	41.8 ± 3.8	39.6 ± 2.5	0.0071	Pain for warm (°C)	44.7 ± 2.8	42.6 ± 1.8	0.003
Vibration (*μ*)	2.14 ± 1.40	1.22 ± 0.27	0.0077	Vibration (*μ*)	3.59 ± 2.65	1.80 ± 0.35	0.0014

QST, quantitative sensory test.

Additionally, we analyzed a subgroup of 15 patients with neuropathologically diagnosed of glioblastoma multiforme. None of these patients had their autoantibodies confirmed in line blotting. There were no statistically significant differences in the electrophysiological studies between patients with glioblastoma multiforme and those with other types of tumors (Table 5).

**Table 5:** Clinical and electrophysiological data from five patients with positive antibodies

	Patient 1	Patient 2	Patient 3	Patient 4	Patient 5	Normal values
Type of tumor	Schwanoma	Astrocytoma anaplasticum	Meningioma pseudomanas	Astrocytoma fibrillare grade II	Ependymoma	
Type of antibody	Anti‐neuro ‐endothelium	Anti‐MAG	Anti‐PCNA	Anti‐GFAP	Anti‐Ro52	
Clinical polyneuropathy	No	Yes	No	No	No	
Peroneal nerve motor conduction velocity (m/sec)	41.8	49.3	52.5	50.0	45.1	49.6 ± 4.1
CVD lower quartile (m/sec)	34.6	38.5	38	33	38.0	40.7 ± 2.5
Median velocity (m/sec)	37.8	42.6	41.1	37.8	40.6	45.0 ± 2.8
CVD upper quartile (m/sec)	39.5	45.3	44.9	41.0	41.7	48.4 ± 2.6
Sural nerve sensory conduction velocity (m/sec)	35.5	0	56.5	51.5	50.0	51.8 ± 4.3

CVD, conduction velocity distribution.

## Discussion

In the European study on PNSs in the PNS Euronetwork Database published in 2010, in 968 patients with defined PNSs, there were no tumors of the central nervous system, only neuroblastoma was diagnosed in seven patients but without defined location. However, sensory neuropathy occurred in 24.3% of the patients, other neuropathies in 8.7%, muscles were involved only in 1.6% of the patients (Giometto et al. 2010). In our study group, we were able to recognize peripheral nerve lesions, with predominantly demyelinating features, not only of sensory, but also of motor fibers, with a tendency to small fiber changes seen in SSR and QST tests. In only three patients was the polyneuropathy clinically obvious; in the others, the symptoms were subclinical. We did not find well‐defined onconeuronal antibodies in any of our patients. We revealed antineural antibodies which in most cases were directed against antigens localized in both the central and peripheral nervous system. Antinucleosome antibodies, which belong to the group of ANA (antinuclear antibodies), are found in all rheumatic diseases, and nucleosomes are thought to be the most relevant autoantigens for antibody production. Antinucleosome antibodies seem to correlate with disease activity, and they have a great reliability as a marker of activity changes in systemic lupus erythematosus. ANA are also found more frequently in patients with different malignancies (Gultekin et al. 1998; Abu‐Shakara et al. 2001; Smeenk 2009; Li et al. 2015). In Heegaard et al.'s study (Heegaard et al. 2012), ANA were detected in 40% of ovarian cancer patients, and in these patients survival periods were significantly shorter than those for ANA‐negative cancer patients. The authors concluded that circulating antibodies are very promising as biomarkers in different malignancies.

Anti‐neuroendothelium antibodies may interact with endothelial cells localized in both the central and peripheral nervous system, for example, in *vasa nervorum*. We are not aware that such a cross reactivity has been evidenced yet. GFAP expression was found in satellite cells of the dorsal root ganglion (Woodham et al. 1989; Siemionow et al. 2009). Thus, cross reactivity seems to be highly probable.

Anti‐MAG, anti‐GFAP, and anti‐neuroendothelium antibodies were identified in our study only by means of indirect fluorescence, however, there are no validated, clinically available/acceptable confirmation tests with “Conformite Europeenne” certificate (CE Mark) that use recombinant antigens for the detection of above‐mentioned antibodies. The detection of anti‐MAG antibodies by means of indirect immunofluorescence was characterized by the 83% sensitivity of and 95% specificity (Caudie et al. 2006). We are not aware about the data on sensitivity and specificity of indirect immunofluorescence for the detection of anti‐GFAP and anti‐neuroendothelium antibodies.

As yet, there are no studies on correlations between primary brain tumors, different circulating antibodies (onconeuronal, antineural), and possible paraneoplastic syndromes. We found only a few case reports (Barisic et al. 2007; Derrett‐Smith and Isenberg 2008; Sanli et al. 2010; Melguizo et al. 2011; Nakano et al. 2013) indicating the possibility of paraneoplastic phenomena in the course of primary brain tumors. Most of these were not clear because of the coadministration of the immunotheraphy. The presented findings do strongly suggest the induction of autoimmunity in patients with primary brain tumors. We managed to identify and confirm five different types of antibodies (anti‐MAG, anti‐GFAP, anti‐PCNA, anti‐Ro52, and anti‐neuroendothelium). Antineural immunity is thought to be often connected with peripheral neuropathy (Tschernatsch et al. 2005; Steck et al. 2013). The electrophysiological findings showed the significant changes of peripheral nerves throughout the patient group, and in five patients with confirmed antineuronal antibodies, the respective electrophysiological results were also outside the normal limits. None of the patients with glioblastoma multiforme had confirmed antineuronal antibodies; in one case, antibodies against nucleosome were seen only in screening. Patients with glioblastoma multiforme had similar electrophysiological changes of peripheral nerves as other patients. These findings suggest that in high‐grade tumors with recent disease onset, we could probably expect other kinds of antibodies. Our patients did not fulfill totally the Graus et al. (2004) diagnostic criteria for PNS, but they could be eventually classified as a possible PNS point 3 (a nonclassical neurological syndrome, no onconeuronal antibodies, and cancer present within 2 years of diagnosis).

Investigations into autoimmunity in patients with primary brain tumors must be continued on a larger patient group, with estimation of a wide range of different antibodies, because paraneoplastic syndromes, as a possible consequence of immunological activation, must be well defined in these cases.

## Conclusions

In patients with primary brain tumors, electrophysiological changes of peripheral nerves associated with the presence of antineural antibodies suggest an autoimmune humoral response, and make the diagnosis of PNS possible.

## Conflict of Interest

All authors declare that they have no financial, nonfinancial, professional, and personal competing interests.
